# Postoperative Functional Recovery After Gastrectomy in Patients Undergoing Enhanced Recovery After Surgery: A Prospective Assessment Using Standard Discharge Criteria

**DOI:** 10.1097/MD.0000000000003140

**Published:** 2016-04-08

**Authors:** Oh Jeong, Seong Yeob Ryu, Young Kyu Park

**Affiliations:** From the Division of Gastroenterological Surgery, Department of Surgery, College of Medicine, Chonnam National University, South Korea.

## Abstract

Enhanced recovery after surgery (ERAS) is increasingly used in several abdominal surgeries to accelerate postoperative recovery and reduce the length of stay. The aim of this study was to investigate the pattern of functional recovery after gastrectomy in patients undergoing ERAS and to analyze factors that affect postoperative recovery.

In all, 168 gastric cancer patients enrolled in a clinical trial evaluating ERAS compliance after gastrectomy were prospectively assessed with respect to postoperative functional recovery using discharge criteria, evaluating 4 major functional outcomes: adequate pain control, ability to mobilize and self-care, tolerance of oral intake, and no abnormal physical findings or laboratory test.

The mean completion time of overall discharge criteria was 5.1 ± 3.2 days. The mean completion time for each dimension were 4.4 ± 0.9 days for adequate pain control, 4.1 ± 0.8 days for ability to mobilize and self-care, 4.3 ± 1.1 days for no abnormal physical signs or laboratory test, and 4.6 ± 1.2 days for tolerance of oral intake. The mean length of stay was 7.2 ± 3.2 days, and readmission rate was 2.4% (n = 4). There was 9.5% (n = 16) of morbidity and no hospital mortality. Female sex (*P* < 0.001) and age (≥65 years; *P* = 0.049) were significantly associated with a slower recovery in tolerance of oral intake, and total gastrectomy was significantly associated with delayed completion of adequate pain control (*P* = 0.003).

Functional recovery after gastrectomy can be achieved after about 5 days in patients undergoing ERAS. Female sex, old age, and total gastrectomy are factors that delay normal functional recovery after gastrectomy.

## Abstract 2

Supplemental Digital Content is available in the text

## INTRODUCTION

Over the past few decades, there have been significant advances in anesthesia/analgesia, operative techniques, and perioperative care in major abdominal surgery.^1^ More recently, multimodal strategies to optimize perioperative care based on scientific evidence have evolved into the concept of “enhanced recovery after surgery” (ERAS).^2^ ERAS aims to shorten the length of hospital stay by accelerating postoperative recovery and reducing surgical stress throughout the course of perioperative care.^3^ In colonic surgery, several randomized trials and meta-analyses have shown that ERAS can effectively reduce the length of hospital stay and decrease complication rates.^4–6^ ERAS also has been successfully implemented for other abdominal procedures, such as liver/pancreatic surgery, esophageal surgery, gastric surgery, bariatric surgery, gynecologic surgery, and emergency surgery.^7^

Discharge criteria refer to predefined clinical conditions that determine readiness for hospital discharge after surgery.^8^ By evaluating fulfillment of discharge criteria, a surgical team makes an objective decision that a patient has recovered sufficiently to be safely managed outside the acute hospital setting. Premature discharge without proper evaluation of postoperative recovery may increase the risk of readmission and mortality.^9^ Therefore, developing standardized criteria is considered valuable in terms of reducing the risk of premature discharge and avoiding unnecessarily lengthy hospital stays. In addition, discharge criteria allow for a more accurate comparison of results between studies investigating the efficacy of interventions that aim to accelerate postoperative recovery. However, as no widely accepted standardized discharge criteria for gastrointestinal surgery exist yet, the criteria used in previous studies were highly variable and poorly defined.^10^

Unlike colonic surgery, ERAS has been less widely used in conjunction with gastric cancer surgery. Recently, some researchers have suggested that ERAS reduces the length of stay by up to 1 to 3 days compared with conventional care in patients undergoing gastrectomy.^11–15^ However, previous studies mostly focused on the length of stay as a primary outcome of the efficacy of ERAS. Actual postoperative recovery in diverse functional dimensions has not been evaluated in patients undergoing ERAS. In the present study, we prospectively investigated the patterns of postoperative functional recovery after gastrectomy in patients undergoing ERAS using standard discharge criteria.

## METHODS

### Patients

Between August 2012 and December 2013, 168 patients were enrolled in a clinical trial that investigated the compliance of ERAS after gastrectomy (ClinicalTrials.gov, NCT01653496). The primary purpose of the study was to evaluate the compliance rates of ERAS elements in patients undergoing gastrectomy, and this study is the secondary analysis of the registered trial. Using these patients, we prospectively assessed postoperative functional recovery using standard discharge criteria after surgery. Inclusion criteria were as follows: patients undergoing gastrectomy for gastric carcinoma, age 18 to 75 years, Eastern Cooperative Oncology Group (ECOG) performance status of 0 to 2, normal hepatic, renal, and hematologic function, and provision of written informed consent. Patients who underwent previous chemotherapy, had other combined organ malignancies, or underwent an operation under emergency conditions were not included. This study was approved by the institutional review board of our institution (Chonnam National University Hwasun Hospital, South Korea). We obtained informed consent from all patients participating in this study.

Preoperative work-up included endoscopy with biopsy and abdominal computed tomography (CT) scan, along with chest radiography, electrocardiogram, and basic blood and pulmonary function testing. Endoscopic ultrasonography, magnetic resonance imaging, or chest CT scan was selectively performed if required. Three experienced gastric surgeons participated in this study. All surgeons were faculties of our department and had 8 to 20 years of experience in gastric cancer surgery. Patients underwent distal or total gastrectomy with regional lymph node dissection (LND) according to Japanese gastric cancer treatment guidelines (2010, ver. 3).^16^ Laparoscopic surgery was indicated for cT1–2N0 tumors, and otherwise open surgery was performed.

Patient data including demographic features, operative procedures, pathologic results, hospital stay, morbidity, and mortality were prospectively collected. Morbidity and mortality were defined as any complications or deaths that occurred within postoperative 30 days or during hospitalization. Postoperative complications were classified as local or systemic depending on whether or not they related to the operation. The severity of complications was graded based on the Clavien–Dindo classification of surgical complications.^17^ Pathologic stages were based on the seventh edition of the Union for International Cancer Control's tumor node metastasis (TNM) classification of malignant tumours.^18^

### ERAS Protocol

The protocol was developed based on the recommendations and guidelines of ERAS, also taking into account the special consideration for gastrectomy and our institutional resources (Supplementary Table S1).^19,20^ A multidisciplinary team consisting of surgeons, anesthesiologists, nurses, and nutritionists participated in the process of protocol development.

Before surgery, patients are informed about the ERAS program and perioperative care plan. Preoperative mechanical bowel preparation is avoided. Patients are permitted to have oral meals until 6 hours before surgery, and a carbohydrate-rich drink is administered 2 hours before surgery begins. In the operating room, a nasogastric tube or abdominal drainage is not routinely administered to patients. Prophylactic antibiotics are given just before skin incision, and no extended dose is used after surgery. During the operation, a normal body temperature is maintained using a warm air blanket. Postoperatively, patient-controlled epidural anesthesia is used for pain control. Venous thromboprophylaxis is performed until hospital discharge using an intermittent pneumatic compression device. Patients are allowed to drink water 6 hours after surgery at will and begin oral intake of food (2/3 bowel of rice porridge and side dishes, 6 times a day) on postoperative day (POD) 1. The urinary catheter is removed as early as POD 1. Fluid overhydration is avoided with restrictive intravenous fluid administration (20 mL/kg/day), which is discontinued on POD 3 if patients tolerate oral intake. Patients are encouraged to actively ambulate from POD 1. The discharge plan is completed on POD 5, assuming patients fulfill all predefined discharge criteria.

### Discharge Criteria

Based on criteria that are commonly indicated in existing literature,^8,10^ we developed 4 main functional criteria and their endpoints that should be met before hospital discharge: adequate pain control, ability to mobilize and self-care, tolerance of oral intake, and no abnormal physical signs or laboratory test (Table 1). Postoperatively, a team of attending physicians and nurses daily assessed completion of each discharge criterion using the checklist from POD 4. Completion of overall discharge criteria was defined as time to fulfill all 4 discharge criteria in a patient. For instance, if a patient fulfilled adequate pain control, ability to mobilize and self-care, and tolerance of oral intake on the POD 4, and no abnormal physical signs or laboratory test on POD 6, the completion of overall discharge criteria was recorded as POD 6. In this study, we analyzed completion of overall criteria, and also each discharge criterion. When all criteria were met, the patient was considered ready for hospital discharge and was ordered to be discharged from the hospital on the next day.

**TABLE 1:**
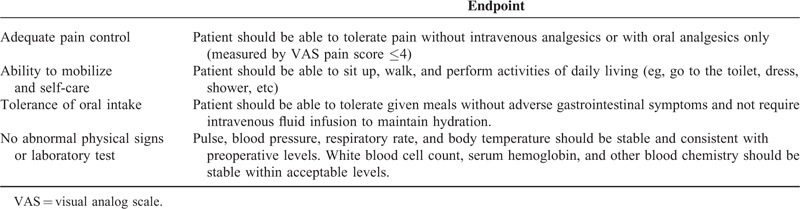
Discharge Criteria

Postoperative pain was assed using visual analog scale (VAS pain score), and completion was defined as patients showed a VAS score of ≤4 without intravenous analgesics or with oral analgesics only. Tolerance of oral intake was assed using patient's diet record, and completion was defined as the ability to consume more than 2/3 of given meal without adverse gastrointestinal symptoms. Ability to mobilize and self-care was evaluated by closely monitoring patient's daily activity.

### Statistical Analysis

Completion times of overall and each discharge criterion were compared between the groups using the Mann–Whitney *U* test. Chi-square or Fisher exact test was used for categorical data, as appropriate. The statistical analyses were performed with SPSS Version 23.0 (SPSS Inc., Chicago, IL); 2-sided *P* values <0.05 were considered statistically significant.

## RESULTS

There were 102 men and 66 women with a mean age of 55.3 ± 10.1 years (Table 2). One hundred fifty-one (89.9%) patients underwent distal gastrectomy and 17 (10.1%) patients underwent total gastrectomy. Laparoscopic surgery was performed on 142 (84.5%) patients. Five (3.1%) patients underwent combined organ resection (2 cholecystectomies, 2 splenectomies, and 1 partial liver resection). The mean operating time was 169 ± 53 minutes. On the final pathologic examination, there were 137 (81.5%) patients with TNM stage I, 21 (12.5%) patients with stage II, 8 (4.8%) patients with stage III, and 2 (1.2%) patients with stage IV.

**TABLE 2:**
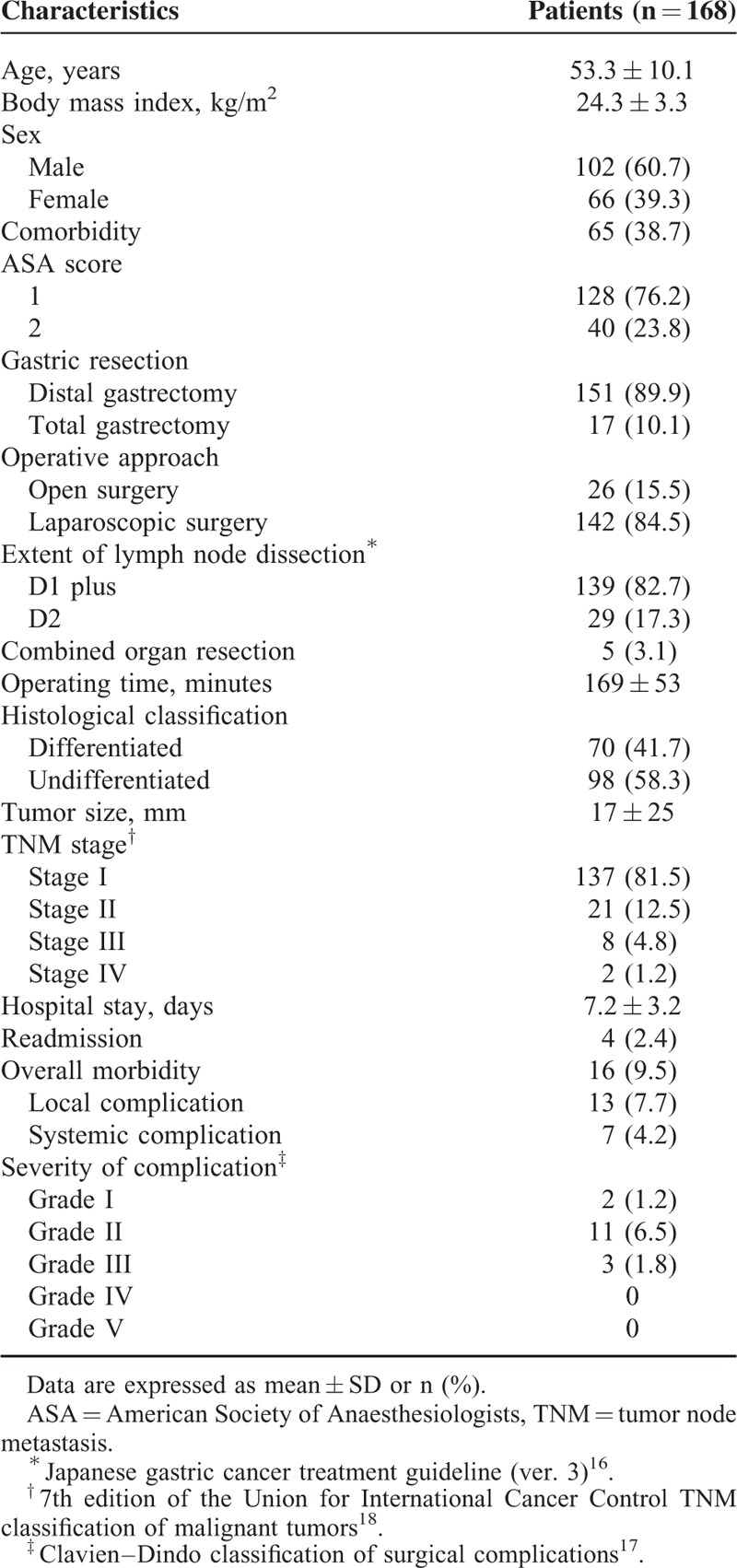
Patient Characteristics

After surgery, 16 (9.5%) patients developed complications, including 13 (7.7%) local and 7 (4.2%) systemic complications. Three (1.8%) patients developed complications of ≥grade III severity. One patient underwent a reoperation due to an intractable luminal bleeding. There was no hospital mortality. Of the local complications, paralytic ileus (n = 4) was the most common, followed by luminal bleeding (n = 3) and intra-abdominal infection (n = 3). Of the systemic complications, urinary tract infection (n = 3) was the most common. The mean length of stay was 7.2 ± 3.2 days, and readmission rate was 2.4% (n = 4, 1 cholecystitis, 1 gastroenteritis, 1 pancreatitis, and 1 gastric stasis).

### Fulfillment of Discharge Criteria

Table 3 shows postoperative fulfillment of discharge criteria in overall patients. Completion of overall discharge criteria were achieved by 100 (59.5%) patients within POD 4; 128 (76.2%) patients within POD 5; and 155 (92.3%) patients within POD 6. The mean completion time of discharge criteria in overall patients was 5.1 ± 3.2 days. There was a significant difference in mean completion time between the patients with and without morbidity (10.4 ± 8.7 vs 4.6 ± 0.9 days; *P* < 0.001). When patients without postoperative morbidity (n = 152) were analyzed separately, 97 (63.8%) patients completed the discharge criteria within POD 4; 123 (80.9%) patients within POD 5; and 147 (96.7%) patients within POD 6.

**TABLE 3:**
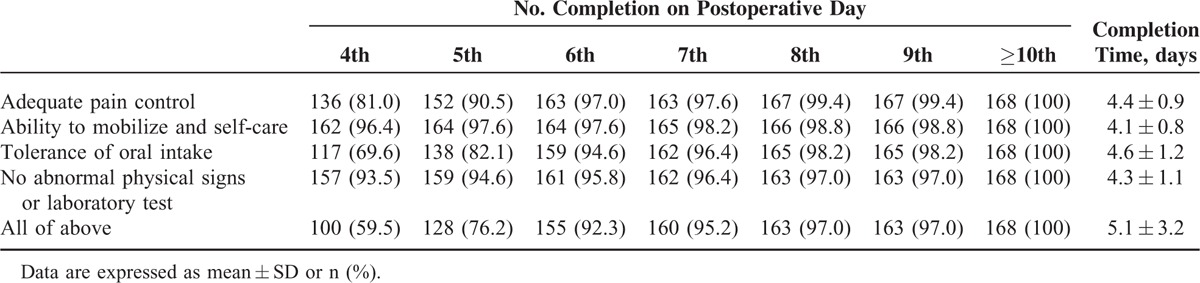
Fulfilment of Discharge Criteria in Overall Patients

The mean completion times of each criterion were 4.4 ± 0.9 days for adequate pain control, 4.1 ± 0.8 days for ability to mobilize and self-care, 4.3 ± 1.1 days for no abnormal physical signs or laboratory test, and 4.6 ± 1.2 days for tolerance to oral intake. Of the discharge criteria, completion of tolerance of oral intake was relatively lower at an early postoperative stage (ie, 69.6% on POD 4 and 82.1% on POD 5) compared with other dimensions.

### Fulfillment of Discharge Criteria in Different Subgroups

To investigate the differences in normal recovery pattern, completion times of each discharge criterion were compared with respect to age, sex, body mass index (BMI), comorbidity, the extent of gastric resection, operative approach, LND, operating time, and tumor stage in patients not experiencing postoperative complications (Table 4).

**TABLE 4:**
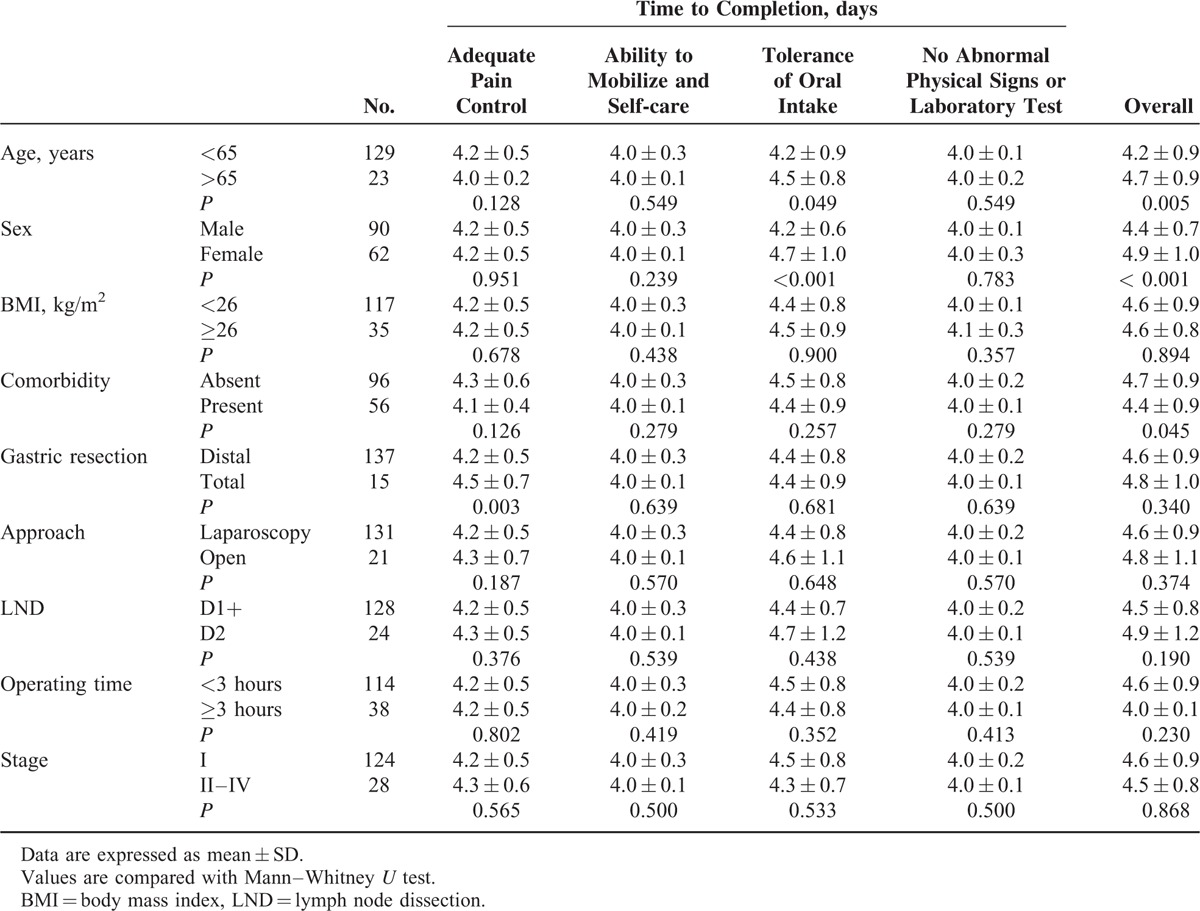
Subgroup Analysis of Completion of Discharge Criteria

Sex (female; *P* < 0.001) and age (≥65 years; *P* = 0.049) were significantly associated with a slower recovery in tolerance of oral intake, which led to delayed completion of overall discharge criteria. Total gastrectomy was associated with delayed completion of adequate pain control (*P* = 0.003), but there was no significant difference in completion of overall discharge criteria compared with distal gastrectomy. The existence of comorbidity was associated with delayed completion of overall discharge criteria (*P* = 0.045). Meanwhile, there were no significant differences in completion of discharge criteria with respect to BMI, operative approach, the extent of LND, operating time, or tumor stage.

## DISCUSSION

This is the first study to investigate postoperative functional recovery after gastrectomy under ERAS protocol. We have demonstrated the recovering patterns of 4 main functional dimensions that constitute minimum physiologic requirements for hospital discharge. We have found that functional recovery meeting the discharge requirement can be achieved in early postoperative period (within 1 postoperative week) in most patients after gastrectomy with ERAS program. Therefore, our study suggests that discharge plan within 1 postoperative week may be reasonable after gastrectomy under ERAS management.

The ERAS protocol aims to reduce the length of hospital stay by accelerating postoperative recovery using evidence-based multimodal strategies.^3^ However, there is concern that premature discharge without proper evaluation of postoperative recovery may increase the risk of readmission and mortality.^9^ Therefore, developing standardized discharge criteria is considered valuable to make a proper discharge decision in ERAS. Also, use of standard discharge criteria may lead to convincing evidence for the studies investigating the efficacy of any interventions aimed at enhancing postoperative recovery.^21^

In our study, patients with preoperative chemotherapy were not included. Preoperative chemotherapy has now become a standard treatment for locally advanced gastric carcinoma worldwide. However, it is not routinely performed in East Asian countries except for selected far advanced cases, and upfront surgery is a primary treatment for most cases. Because of relatively weak and vulnerable condition in patients with preoperative chemotherapy, we did not enroll those patients in our conventional ERAS program. We believe perioperative care program in those patients may require a tailed approach different from conventional ERAS program. Therefore, postoperative functional recovery in those patients is expected to differ from that of upfront surgery, and it will need further investigation.

There are no standard discharge criteria to indicate readiness for hospital discharge after abdominal surgery. In a review of 156 colorectal studies, it was found that tolerance of oral intake, return of bowl function, adequate pain control, and adequate mobility were the most frequently used discharge criteria in the literature.^10^ Meanwhile, an ERAS research group suggested 5 discharge criteria for colorectal surgery: good pain control with oral analgesia, tolerance of solid food, no intravenous fluid treatment, ability to be independently mobile, and willingness to go home.^22^ However, no specific endpoints for these criteria were described in detail. In 2012, an international consensus was made by 15 experts from different countries regarding hospital discharge criteria for patients undergoing colorectal surgery.^8^ In their report, the experts agreed on 5 criteria with specific endpoints to determine readiness for hospital discharge: tolerance of oral intake, recovery of lower gastrointestinal function, adequate pain control with oral analgesia, ability to mobilize and self-care, and no evidence of complications or untreated medical problems. These criteria are also considered generally acceptable for other gastrointestinal surgeries with minor modification. However, the clinical use of discharge criteria may vary depending on differences in the hospital system and available healthcare resources.

In reality, there is often some delay in actual hospital discharge after a patient fulfills the discharge criteria. The causes of delay can be the patient's demands, requirements by the hospital system, or surgeon's discretion. In Korea, patients have less economic burden with longer hospital stay because of the low medical cost for hospital stay. In our experience, 72 (43%) patients stayed 1 to 3 more days at their demand after they were informed to be discharged from the hospital. Consequently, the actual length of stay was 7.2 days, compared with 5.2 days to complete the discharge criteria. Interestingly, however, patients with delayed hospital discharge showed no significant difference in readmission rate compared with those who were discharged on the designated date (*P* = 0.574). Similarly, Maessen et al^23^ reported a median of 2 days of delay in hospital discharge after fulfilling discharge criteria for nearly 90% of patients after colonic surgery. These suggest that organized efforts are also essential to maximize the intended effect of ERAS on reducing the length of hospital stay.^24^ Advanced postdischarge care service, such as home healthcare, close outpatient monitoring, or provision of easy access to the clinic, are good solutions to enable safe discharge of patients on the day of functional recovery.

In the present study, old age and female sex were significantly associated with delayed completion of tolerance of oral intake, and total gastrectomy significantly delayed completion in adequate pain control. A possible explanation for this is that female and older patients are more sensitive to gastrointestinal discomfort when they start eating, and tend to more gradually and carefully increase the food amount compared with male or younger patients. Meanwhile, an interesting finding was that fulfillment of discharge criteria was not significantly different by the type of operative approach (laparoscopy vs open). This finding is in contrast with previous reports showing reduced length of stay by laparoscopic gastrectomy.^25^ We think that this is probably because functional recovery of the 2 surgery groups was compared with using same ERAS protocol including diet and pain control. Meanwhile, Basse et al^26^ have shown that there was no significant difference in hospital stay between open and laparoscopic colonic surgery when same ERAS was equally applied to both surgery groups. Because of small number of open surgery in our study, the difference in functional recovery between 2 operative approaches requires further investigation. However, it should be emphasized that the efficacy of laparoscopic surgery needs to be further validated using outcomes that better indicate actual functional recovery.

There are some limitations to this study. First, the discharge criteria used were primarily based on consensus for colonic surgery and have not been validated for gastric cancer surgery. Currently, there are no validated standard discharge criteria for gastrectomy, and those tools are usually regarded as acceptable for other types of gastrointestinal surgery. Second, this study may have some selection bias due to relatively strict inclusion and exclusion criteria (ie, exclusion of poor ECOG status or very old age). However, this study will be helpful to understand the normal recovery process in surgically fit gastric cancer patients with elective surgery. The low number of upper gastric cancer also may limit the generalizability of our results, which requires further investigation in large patients. Third, assessment of functional recovery using discharge criteria was limited by the somewhat arbitrary nature of its endpoints. For instance, a decision regarding a patient's tolerance of oral intake can be ambiguous without consideration of the amount of the meal that the patient desires to consume. Finally, this study did not provide data comparing fulfillment of discharge criteria between patients receiving ERAS and a conventional care group. Based on our results, we are now planning a randomized controlled trial to investigate the efficacy of ERAS using discharge criteria.

In conclusion, a proper care plan requires better understanding about the postoperative recovery process in surgical patients. This study investigated the pattern of postoperative functional recovery after gastrectomy under ERAS. We have found that adequate functional recovery for hospital discharge can be achieved in early postoperative period (within 1 postoperative week) after gastrectomy with ERAS program. Finally, the efficacy of ERAS on postoperative recovery needs to be more objectively evaluated rather than using length of stay alone.

## Supplementary Material

Supplemental Digital Content
