# Supplementary material for: Postoperative Functional Recovery After Gastrectomy in Patients Undergoing Enhanced Recovery After Surgery: A Prospective Assessment Using Standard Discharge Criteria
Source: Medicine (Baltimore). 2016 Apr 8;95(14):e3140. doi: 10.1097/MD.0000000000003140 (PMC4998752; doi:10.1097/MD.0000000000003140)
Supplement: Supplemental Digital Content [file medi-95-e3140-s001.doc]

**SUPPLEMENTARY MATERIAL**

**Table S1 ERAS protocol**

| Element | Practice |
| --- | --- |
| **Preoperative** | |
| Patient education | Educate ERAS program: health information, ERAS protocol, discharge plan, etc. |
| Preoperative bowl preparation | No routine use of mechanical bowl preparation using laxative before surgery |
| Preoperative fasting | Allow oral meal intake until 6 hours before surgery |
| Intraoperative | |
| Nasogastric tube/abdominal drainage | No routine use of nasogastric tube or abdominal drainage during surgery |
| Carbohydrate-rich drink | Administer 200 to 400 ml of carbohydrate-rich drink 2 hours before surgery |
| Intraoperative normothermia | Maintain normal body temperature using warm-air circulating blanket during surgery |
| Prophylactic antibiotics | Single-dose antibiotics before skin incision without extended postoperative use |
| **Postoperative** | |
| Pain control | Patient-controlled epidural analgesia with additional non-opioid injection on demand |
| Thromboprophylaxis | Intermittent pneumatic compression device until hospital discharge |
| Oral nutrition | Allow oral water intake 6 hours after surgery and start early oral nutrition from postoperative day 1 |
| Intravenous fluid administration | Avoid over-hydration and restricted fluid administration (20ml/kg/d) during first 3 postoperative days |
| Early ambulation | Encourage early active ambulation from postoperative day 1 (more than 2hr/d) |
| Urinary catheter removal | Remove urinary catheter as early as postoperative day 1 |
| Hospital discharge | Evaluate patient recovery with discharge criteria in order to determine discharge plan |

ERAS, enhanced recovery after surgery.
